# Mortality Prediction of the CHA_2_DS_2_-VASc Score, the HAS-BLED Score, and Their Combination in Anticoagulated Patients with Atrial Fibrillation

**DOI:** 10.3390/jcm9123987

**Published:** 2020-12-09

**Authors:** Doralisa Morrone, Sonja Kroep, Fabrizio Ricci, Giulia Renda, Giuseppe Patti, Paulus Kirchhof, Ling-Hsiang Chuang, Ben van Hout, Raffaele De Caterina

**Affiliations:** 1 Division of Cardiology, Department of Surgical, Medical and Molecular Pathology and Critical Care Medicine, University of Pisa, 50124 Pisa, Italy; doralisa.morrone@unipi.it; 2 Pharmerit—An OPEN Health Company, 3068 AV Rotterdam, The Netherlands; skroep@pharmerit.com (S.K.); ling-hsiang.chuang@york.ac.uk (L.-H.C.); bvanhout@pharmerit.com (B.v.H.); 3 Institute of Cardiology and Center of Excellence on Aging, G. d’Annunzio University, 66100 Chieti-Pescara, Italy; fabrizioricci@hotmail.it (F.R.); grenda@unich.it (G.R.); 4 Fondazione VillaSerena per la Ricerca, Città Sant’Angelo, 65013 Pescara, Italy; 5 Department of Thoracic and Cardiovascular Diseases, University of Eastern Piedmont, 28100 Novara, Italy; giuseppe.patti@uniupo.it; 6 University of Birmingham Institute of Cardiovascular Sciences, University of Birmingham, UHB and SWBH NHS Trusts, Birmingham B15 2TT, UK; p.kirchhof@bham.ac.uk; 7 Heart and Vascular Center, Hamburg University, 20251 Hamburg, Germany; 8 School of Health and Related Research (ScHARR), The University of Sheffield, Sheffield S10 2TN, UK

**Keywords:** atrial fibrillation, morbidity, mortality, risk scores, CHA_2_DS_2_-VASc score, HAS-BLED score

## Abstract

Background and Objectives: Atrial fibrillation (AF) is associated with increased mortality, predictors of which are poorly characterized. We investigated the predictive power of the commonly used CHA_2_DS_2_-VASc score (congestive heart failure, hypertension, age ≥ 75 years [doubled], diabetes, stroke/transient ischemic attack/thromboembolism [doubled], vascular disease [prior myocardial infarction, peripheral artery disease, or aortic plaque], age 65–75 years, sex category [female]), the HAS-BLED score (hypertension, abnormal renal/liver function, stroke, bleeding history or predisposition, labile international normalized ratio [INR], elderly [age ≥ 65 years], drugs/alcohol concomitantly), and their combination for mortality in AF patients. Methods: The PREvention oF thromboembolic events—European Registry in Atrial Fibrillation (PREFER in AF) was a prospective registry including AF patients across seven European countries. We used logistic regression to analyze the relationship between the CHA_2_DS_2_-VASc and HAS-BLED scores and outcomes, including mortality, at one year. We evaluated the performance of logistic regression models by discrimination measures (C-index and DeLong test) and calibration measures (Hosmer and Lemeshow goodness-of-fit and integrated discrimination improvement (IDI), with bootstrap techniques for internal validation. Results: In 5209 AF patients with complete information on both scores, average one-year mortality was 3.1%. We found strong gradients between stroke/systemic embolic events (SSE), major bleeding and—specifically—mortality for both CHA_2_DS_2_-VASc and HAS-BLED scores, with a similar C-statistic for event prediction. The predictive power of the models with both scores combined, removing overlapping components, was significantly enhanced (*p* < 0.01) compared to models including either CHA_2_DS_2_-VASc or HAS-BLED alone: for mortality, C-statistic: 0.740, compared to 0.707 for CHA_2_DS_2_-VASc or 0.646 for HAS-BLED alone. IDI analyses supported the significant improvement for the combined score model compared to separate score models for all outcomes. Conclusions: Both the CHA_2_DS_2_-VASc and the HAS-BLED scores predict mortality similarly in patients with AF, and a combination of their components increases prediction significantly. Such combination may be useful for investigational and—possibly—also clinical purposes.

## 1. Introduction

Atrial fibrillation (AF) is common, and is associated not only with high incidences of stroke, thromboembolism and disabilities, but also with significant mortality [1,2]. Treatment guidelines recommend considering both thromboembolic and bleeding risks when prescribing anticoagulation for stroke prevention in AF [1,2]. The CHA_2_DS_2_-VASc score (congestive heart failure, hypertension, age ≥ 75 years [doubled], diabetes, stroke/transient ischemic attack/thromboembolism [doubled], vascular disease [prior myocardial infarction, peripheral artery disease, or aortic plaque], age 65–75 years, sex category [female]) has been developed as a clinical risk score in patients with AF to predict the risk of stroke [3], and is now adopted in most widely used guidelines to assess such risk [1,2]. In some studies, the CHA_2_DS_2_-VASc score has also been used beyond its original purpose and disease populations. The score has been shown to predict outcomes broader than ischemic stroke, for instance thromboembolic events and death, in non-AF patient populations, such as patients with ischemic heart disease [4], diabetes [5], heart failure [6], in non-AF community patients [7], or in unselected patients [8]. The HAS-BLED score (hypertension, abnormal renal/liver function, stroke, bleeding history or predisposition, labile international normalized ratio [INR], elderly [age ≥ 65 years], drugs/alcohol concomitantly) was developed to predict bleeding amongst AF patients [9]. It is still widely used, although now de-emphasized due the lesser relevance of bleeding considerations in isolation to determine therapy for AF [1,2].

Due to the overlapping risk factors between CHA_2_DS_2_-VASc and HAS-BLED scores, both appear to predict adverse events of any kind. The compared performances of the two scores and their potential combination for the prediction of mortality have not been previously evaluated, and might be of interest due to the wide adoption of these scores. The aim of the current study was to investigate such predictive power in patients with AF.

## 2. Methods

Individual patient data were pooled from the PREvention oF thromboembolic events—European Registry in Atrial Fibrillation (PREFER in AF), a prospective registry of 7243 AF patients from 461 hospitals and 7 European countries (Austria, France, Germany, Italy, Spain, Switzerland, and the United Kingdom) conducted between January 2012 and January 2013, with 1-year follow-up. Details of the PREFER in AF Registry, including main findings at enrolment and definitions used, have been published [10]. For the purpose of this study, we focused on the following outcomes: ischemic stroke and or systemic embolic events (SEE), major bleeding and all-cause mortality.

### 2.1. Registry Data and Patients’ Population

Patients were included in the PREFER in AF registry if they were at least 18 years of age, gave written informed consent for participation, and had a history of AF documented by electrocardiography or by an implanted pacemaker or defibrillator within the preceding 12 months. Patients were consecutively enrolled with no explicit exclusion criteria in order to avoid selection biases. Patients’ characteristics, including demographic and clinical data, information on risk factors and treatment strategies, were collected at baseline and entered into an electronic case-report form with on-site verification of source data in approximately 5% of the sites. The study management was overseen by a scientific Steering Committee, as published [10]. Baseline data were collected between January 2012 and January 2013. A total of 7243 patients were enrolled in 461 centers from seven European countries (Austria, France, Germany, Italy, Spain, Switzerland, and the U.K.). For regional comparisons, Austria, Switzerland, and Germany were combined into one pre-specified region labelled “DACH”. Forty-two percent of enrolling physicians were office-based, and 53% were hospital-based; 89% were cardiologists. After baseline inclusion, patients were followed-up for one year, recording information on clinical events, clinical characteristics, patterns of prescriptions and patients’ adherence to guidelines, quality of life and treatment satisfaction.

One year after the baseline enrolment, patients underwent a follow-up visit (12 ± 2 months after baseline visit). As for the baseline visit, all data were captured through an electronic case report form (eCRF) including a wide range of plausibility checks for the entered variables. In addition, on-site source data verification was performed in approximately 5% of the sites. The study management was executed through a contract research organization (SSS International Clinical Research GmbH, Munich, Germany). The study management was overseen by a scientific steering committee. Considered variables were the same as the enrollment visit, including demographic data, data about AF incidence and type, heart rate and symptoms, risk factors and comorbidities, thromboembolic risk evaluated by CHADS_2_ and CHA_2_DS_2_-VASc score, bleeding risk from the HAS-BLED score, history of significant clinical events and hospitalizations, treatment for AF, and anticoagulation quality evaluated by the last 3 INR values prior to follow-up visit.

Patients who completed both CHA_2_DS_2_-VASc and HAS-BLED risk scores at baseline and at the one-year follow-up were included in the current study. The CHA_2_DS_2_-VASc score ranges between 0 and 9, with a higher score indicating a higher risk of stroke. HAS-BLED also ranges between 0 and 9, with a higher score indicating a higher risk of bleeding. For the purpose of this analysis, the CHA_2_DS_2_-VASc and HAS-BLED risk scores at study entry were used. For the analysis of their combination, overlapping components were used only once. Because of some differences in the definition of hypertension (blood pressure consistently above 140/90 mmHg or treated hypertension on medication in the CHA_2_DS_2_-VASc score [3]; uncontrolled, >160 mmHg systolic in the HAS-BLED score [9]), the more inclusive definition of hypertension in the CHA_2_DS_2_-VASc score was retained for the combination score, in this case therefore ranging between 0 and 12. Sensitivity analyses were conducted to assess whether medication type had an effect on the model performance.

### 2.2. Outcomes

The combined endpoint of ischemic stroke and/or SEE was defined as ischemic stroke, transient ischemic attack (TIA) (“a small ischemic stroke”), arterial embolism, venous thromboembolic event, or pulmonary thromboembolism event during the follow-up. Major bleeding was defined, according to the definition of the International Society on Thrombosis and Haemostasis (ISTH), as gastrointestinal bleeding, intracerebral bleeding, or other life-threatening or major bleeding occurring during the follow-up [11]. Estimates of death rates relied on spontaneous reporting. Data were extracted from the comments section of the electronic case-report form and then verified with each site to gain more information about their validity.

### 2.3. Statistical Methods

Descriptive statistics are presented here as frequencies and percentages (n, %) for categorical variables, and as means and standard deviations (SDs) for continuous variables. The difference in baseline characteristics between the study sample and non-study sample were tested, using the Chi-squared test, the rank sum test, or the *t*-test as appropriate.

Logistic regression was used to separately analyze the relationships between the three outcome events (i.e., stroke/SSE, major bleeding and mortality) and the CHA_2_DS_2_-VASc and HAS-BLED scores. For each outcome, five analyses were conducted, evaluating different sets of explanatory variables. The first two analyses evaluated the predictive power of the CHA_2_DS_2_-VASc scores. One analysis included the total risk score as a continuous explanatory variable (ranging from 0 to 9), while the second analysis evaluated the individual items of the CHA_2_DS_2_-VASc score [3] as explanatory variables, which were treated as dichotomous variables [i.e., congestive heart failure (or left ventricular systolic dysfunction), hypertension: blood pressure consistently above 140/90 mmHg (or treated hypertension on medication), age: ≥75 years, age 65–74 years, diabetes mellitus, prior stroke or transient ischemic attack or thromboembolism, vascular disease (i.e., peripheral artery disease, myocardial infarction, aortic plaque), female sex]. The third and fourth analyses considered the HAS-BLED score [9], with one analysis including the total risk score only as a continuous exploratory variable (ranging from 0–9), and a second analysis considering individual components of the HAS-BLED scores [9] as dichotomous variables (i.e., hypertension, abnormal renal/liver function, stroke, bleeding history or predisposition, labile international normalized ratio, age ≥ 65 years, drugs/alcohol concomitantly). For the final analysis, the combination of individual items of the CHA_2_DS_2_-VASc and HAS-BLED scores were used as explanatory variables (dichotomously) rather than a combination of the total score, excluding double reporting of overlapping risk items. The confidence intervals of the coefficients for each logistic regression model were corrected for bias and skewness in the distribution of the bootstrap estimates, for which the average was taken over 100 repetitions, obtained by bootstrap with resampling.

The performance of each model was evaluated by discrimination and calibration [12]. To evaluate discrimination, we used the C-index (area under the curve, AUC) using bootstrap to obtain bias-corrected standard errors, after which the null-hypothesis of no differences between the C-indexes of the models was tested using the Wald test. In addition, we evaluated the integrated discrimination improvement (IDI) [13]. We assessed calibration, referring to the agreement between observed number of events and predicted probability of the occurrence of these events [14], using the Hosmer and Lemeshow goodness-of-fit test.

We performed all analyses with the STATA statistical software (StataCorp. 2017. Release 15. College Station, TX, USA).

## 3. Results

### 3.1. Patient Characteristics

In the original PREFER in AF population, of the 7243 patients, age (mean ± SD) was 71.5 ± 11 years, 60.1% were male, and the CHA_2_DS_2_VASc score was 3.4 ± 1.8. Thirty percent of the patients had paroxysmal, 24.0% persistent, 7.2% long-standing persistent, and 38.8% permanent AF. Oral anticoagulation (OAC) was used in most patients: 4799 patients (66.3%) received a vitamin K antagonist (VKA) as mono-therapy, 720 patients (9.9%) a combination of VKA and antiplatelet agents (AP), and 442 patients (6.1%) received a new oral anticoagulant drugs (NOAC). AP alone was given in 808 patients (11.2%) and there was no antithrombotic therapy in 474 patients (6.5%). Amongst 5209 patients who had complete information for both the CHA_2_DS_2_VASc and HAS-BLED scores at baseline, the mean age was 71.8 ± 10.5 years; 3145 subjects (60.4%) were male. This subsample of 5209 patients was representative of the whole sample population published in the registry [10] across a wide spectrum of baseline variables, including comorbidities. Patients’ characteristics at baseline are shown in Table 1. Stroke risk was high (mean CHA_2_DS_2_VASc total score of 3.4 ± 1.8), with a total score ranging between 2 and 5 in the majority of patients (>70%). Bleeding risk had a total score ranging from 1 to 3 in >80% of cases, and a mean HAS-BLED total score of 2.0 ± 1.1. There was a different representation of the type of AF: permanent AF was found more frequently, accounting for 2070 patients (40%); long-standing persistent AF was the least represented category, with only 391 patients (7.52%); and paroxysmal and persistent AF accounted for 1499 and 1239 patients (29% vs. 24%), respectively. In terms of treatment, the majority of patients (3548, 68%) were on treatment with a vitamin K antagonist (VKA) at baseline, and with a small number (305 patients, 6%) taking a non-vitamin K antagonist oral anticoagulant (NOAC).

The majority of the non-study sample (n = 2034 patients, excluded when no risk scores were available) had similar baseline characteristics as patients included in the study sample, with the exception of chronic kidney disease (CKD), AF type at baseline, treatment with NOAC/VKA/antiplatelet agents, and age. CKD was more prevalent in the study sample compared to the non-study sample (study vs. non-study sample: 13.8% vs. 11.2%), while paroxysmal AF was more prevalent in the non-study sample (study vs. non-study sample: 28.8% vs. 33.0%). Patients were older in the study sample (study vs. non-study sample: 71.8 vs. 70.7 years). Finally, baseline treatment of VKA was more prevalent in the study sample, while NOAC and antiplatelet baseline treatment were more prevalent in the non-study sample.

### 3.2. Outcome Distributions

**Figure 1:**
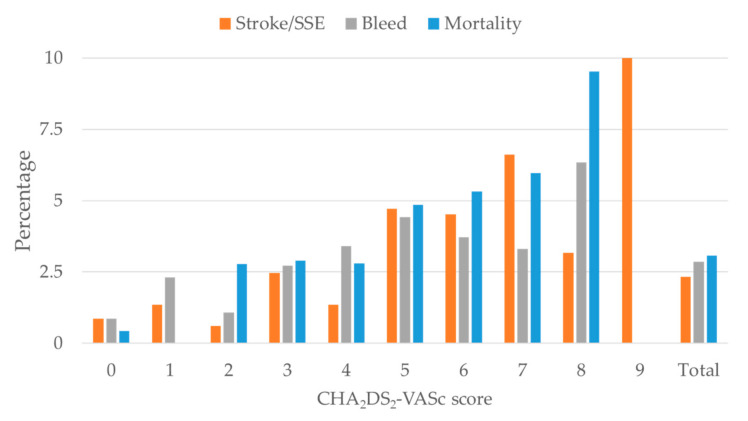
Outcomes (stroke/systemic embolic events (SSE), bleeding and mortality) by CHA_2_DS_2_-VASc risk score in the present study.

**Figure 2:**
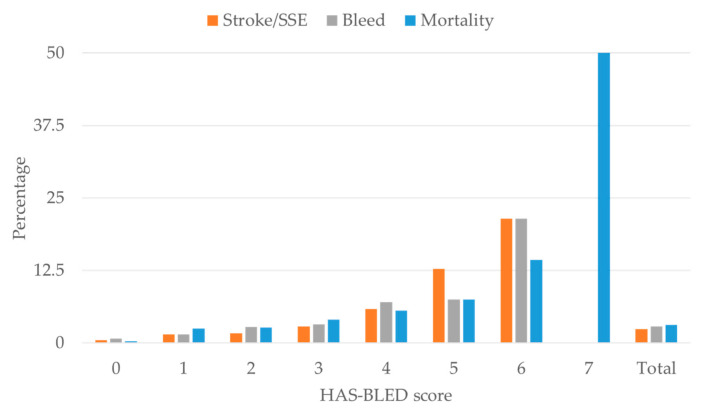
Outcomes (stroke/systemic embolic events (SSE), bleeding and mortality) by HAS-BLED risk score in the present study.

Rates of stroke/SEE and major bleeding at the one-year follow-up were 2.3% (122 patients) and 2.9% (149 patients), respectively. At one year, 3.1% of patients died (160 out of 5209). The percentage of patients with the examined outcomes (i.e., stroke/SSE, major bleeding, and death) in each risk score category of CHA_2_DS_2_VASc and HAS-BLED, respectively, are shown in Figure 1 and Figure 2. There was a strong gradient between the outcome frequency and risk scores: overall, higher risk scores were both positively correlated with the occurred outcomes. However, there were very few events in the highest score category, with only 10 patients having a CHA_2_DS_2_-VASc total score of 9, and only 2 patients with a HAS-BLED total score of 7 (none reported a HAS-BLED total score of 8 or 9).

### 3.3. Logistic Regression Analysis Results

#### 3.3.1. CHA_2_DS_2_-VASc Risk Score

The predictive values of the CHA_2_DS_2_-VASc risk score for each outcome event are shown in Table S1.

Amongst the models including only the total risk score of CHA_2_DS_2_-VASc as an explanatory variable, only the model predicting major bleeding had a good fit (*p*-value > 0.05), as indicated by the Hosmer–Lemeshow goodness-of-fit test (*p* = 0.014, 0.269 and 0.039 for stroke/SSE, major bleeding, and mortality, respectively). As suggested by the C-statistic, the predictive ability was highest for the model predicting stroke/SSE (0.584; 95% CI 0.536–0.637), followed by the model predicting mortality (0.559; 95% 0.513–0.604) and the model predicting major bleeding (0.520; 95% CI 0.478–0.567).

The models with individual components of the CHA_2_DS_2_VASc risk score presented a better fit according to the Hosmer–Lemeshow goodness-of-fit test (with the exception of the model predicting mortality) and an improved predictive ability compared to the model based on the total score alone. Components of the scores assessed here and showing a statistically significant coefficient were different across models predicting different outcomes. For instance, for predicting mortality, congestive heart failure, hypertension, age >75 years, and diabetes mellitus were statistically significant, whereas in predicting stroke/SSE, only the terms congestive heart failure and stroke/TIA had a statistically significant coefficient.

#### 3.3.2. HAS-BLED Risk Score

For the models including only the total risk score of HAS-BLED as an explanatory variable, all models had a good fit (*p*-value > 0.05), as indicated by the Hosmer–Lemeshow goodness-of-fit test (*p* = 0.432, 0.594 and 0.250 for stroke/SSE, major bleeding, and mortality, respectively). Evaluating the models with individual components, the Hosmer–Lemeshow goodness-of-fit test indicated a good fit for all models as well (Table S2). The models with individual components were all compared to models with total scores (C-statistics were significantly improved, *p* < 0.05 for all models). However, items with statistically significant coefficients varied depending on the outcome of interest; for instance, history of bleeding was statistically significant for predicting major bleeding but was not statistically significant for predicting mortality or stroke/SSE (Table S2).

#### 3.3.3. CHA_2_DS_2_-VASc and HAS-BLED Combined

Combining the individual components of the CHA_2_DS_2_VASc and HAS-BLED risk scores with the exclusion of duplicated items generated the most favorable predictive results. All models had a good fit, indicated by the lowest value of the Hosmer goodness-of-fit test (*p*-value > 0.05) with a C-statistic of 0.731 (95% CI 0.681–0.778), 0.702 (95% CI 0.659–0.747) and 0.740 (95% CI 0.699–0.780) for stroke/SSE, major bleeding, and mortality, respectively (Table 2). For predicting stroke/SSE, statistically significant items included congestive heart failure, stroke/TIA, abnormal liver function, labile INR, and drugs; for predicting major bleeding, statistically significant items included age >75 years, vascular disease, abnormal renal and liver function, bleeding and alcohol consumption; and for predicting mortality, statistically significant items included congestive heart failure, hypertension, age >75 years, abnormal renal and liver function.

**Figure 3:**
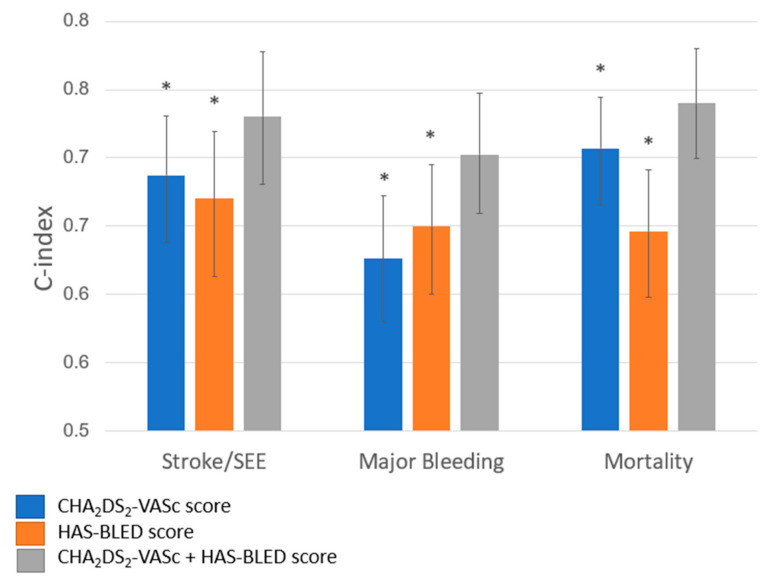
C-index of logistic models by outcome and predictor items risk scores; * denotes a C-index of the specific score significantly different (*p*-value < 0.05) from C-index of the combined score.

The predictive performance of the models combining the CHA_2_DS_2_VASc and HAS-BLED components compared to the CHA_2_DS_2_-VASc individual component models alone was enhanced for the prediction of all outcomes (Table 3 and Figure 3). For mortality, the C-statistic showed a significant improvement (0.707 vs. 0.740, *p* = 0.005). Although the improvement in sensitivity was small, it was significant (IDI: 1.79%; *p* < 0.001). Similar results were demonstrated in models predicting stroke and major bleeding, with the largest improvements demonstrated in the model predicting stroke/SSE.

The improvement of the models with the CHA_2_DS_2_VASc and HAS-BLED combined components over the HAS-BLED individual component models alone was significant in all models in terms of C-statistic and IDI (Table 3 and Figure 3). Moreover, we found small but significant improvement in sensitivity for all outcomes

We found no heterogeneity in analyzing data restricted to patients on NOACs (Table S3). Details on the analyses broken down for AF type (paroxysmal, persistent, long-standing persistent, permanent) are reported in Table S4. We found numerical improvements of the C-statistic in all types of AF, although the achievement of statistically significant improvements was limited by the limitations of sample sizes.

## 4. Discussion

This study has three new major interesting findings: 1. Mortality was higher than the occurrence of stroke/SSE in the patient population of this nearly-contemporary registry with mostly anticoagulated AF patients; 2. Both the CHA_2_DS_2_VASc and the HAS-BLED risk scores were reasonably good predictors of stroke/SSE and major bleeding, but they were also good predictors of mortality; 3. A combination of items included in the two scores significantly improved prediction of death. These results may have practical implications for the prediction of adverse events in patients with AF.

AF is the most common type of heart rhythm disorder and is associated with a 1.5- to 1.9-fold higher risk of death in part due to the strong association with thromboembolic events. Since data from the Framingham heart study, AF has been known to be an independent risk factor for stroke [15]: therefore a major management decision in AF is determining the risk of stroke and appropriate antithrombotic treatment weighted against the risk of serious bleeding. The CHA_2_DS_2_-VASc and HAS-BLED risk scores are the most widely used algorithms to determine the yearly thromboembolic risk and to predict bleeding [16]. They were developed to predict stroke/SSE and bleeding in non-anticoagulated patients, and not developed to predict mortality, which is yet a numerically relevant endpoint in AF even when the risk of stroke/SSE is abated by anticoagulation. Determining, therefore, the comparative performances of these scores in anticoagulated patients not only for stroke/SSE and bleeding, but also for mortality, is new and medically relevant.

### 4.1. Previous Study Comparisons: Scores for Stroke vs. Scores for Bleeding

Previous data from the AMADEUS trial data base in AF patients anticoagulated with either idraparinux or VKAs demonstrated that the CHADS_2_ and CHA_2_DS_2_-VASc scores (used for stroke risk assessment) could be used to predict serious bleeding, comparing against a score—HAS-BLED—intended for bleeding assessment [17]. In a subsequent comparison of stroke risk scores (CHADS_2_ and CHA_2_DS_2_-VASc) and three bleeding risk scores [HAS-BLED, Outcomes Registry for Better Informed Treatment of Atrial Fibrillation (ORBIT), and AnTicoagulation and Risk factors In Atrial fibrillation (ATRIA)] from a large administrative database of NOAC-treated patients in routine clinical practice, CHADS_2_, CHA_2_DS_2_-VASc, HAS-BLED, ORBIT and ATRIA scores had similar performances in predicting major bleeding, highlighting that patients at a high risk of stroke are also at high risk of bleeding [18]. Thus, previous data concur with the present findings, indicating that current risk scores, also because of overlapping components, do not have a high discriminating capacity for stroke/SSE vs. bleeding events.

### 4.2. Previous Studies for Mortality Prediction in Atrial Fibrillation

We carried out a comprehensive literature search to investigate published articles regarding the prediction of death through clinical scores derived for AF. We retrieved 25 published articles referring to mortality prediction (Table S5), Whenever in AF, all these studies referred to anticoagulated patients, a setting different from the original one used to derive the CHA_2_DS_2_-VASc score [3]. In several instances, causes of death in AF appeared unrelated to thromboembolism or bleeding. For example, in anticoagulated AF patients with a median CHA_2_DS_2_-VASc score of 4, Gallego et al. showed that more deaths (4.5/100 patient-years) occurred for causes other than a thromboembolic (only 1.3 patient-years) or a hemorrhagic event (0.4 patient-years). Here, the HAS-BLED score, besides bleeding, predicted adverse events, including all-cause and cardiovascular mortality [19].

Several studies out of AF have shown that scores such as HAS-BLED [20] and CHA_2_DS_2_-VASc [4,5,21,22,23] predict adverse events other than stroke and bleeding, including death. Some previous studies, such as the one from the GARFIELD-AF registry [24], have reported a better prediction of events than the two above scores. Previous literature converges in demonstrating death as a numerically frequent outcome in anticoagulated AF patients, more than thromboembolic or bleeding events; and that scores are general markers of risk, including the risk of death. No previous report, however, has addressed the possibility of predicting risk through a combination of CHA_2_DS_2_-VASc and HAS-BLED. This would have medical relevance due to the current widespread adoption of these scores.

### 4.3. Added Value of the Present Study from the PREFER in AF Registry

The PREFER in AF registry informed on AF patient profiles in a real-world clinical context, helping to identify specific at-risk patient groups, including those with comorbidities that predispose them to thrombotic events. The current study deriving from that registry is the first that compares and combines a stroke risk score with a bleeding score in order to give the best predictive value on mortality. In line with previously published studies, our report shows strong gradients between the CHA_2_DS_2_-VASc and the HAS-BLED risk scores and outcomes examined (stroke/SSE, major bleeding and death). Here, the CHA_2_DS_2_-VASc score predicts mortality, in addition to stroke/SSE, better than the HAS-BLED. Our study also confirms the previous literature in showing that statistical predictions are in any case sub-optimal, in the light of the moderately good C-statistic. The risk of stroke/SSE and major bleeding in our study population is slightly higher than in other publications studying the PREFER in AF registry (see references listed in Table S5). This is likely due to the higher risk of the population included and perhaps also because of the compression of risk of stroke and major bleeding observed with the introduction of the NOACs. Our study, in particular, highlights the different weight of the individual components of both scores to predict future death: here, the hierarchy of predictive weight included congestive heart failure, hypertension, age >75 years, and diabetes among the CHA_2_DS_2_-VASc components; whereas hypertension, liver function and age >65 years were the strongest predictors of mortality in the HAS-BLED risk score. The different weight of individual components in risk prediction for various outcomes is evidently diluted when attributing similar weights (1 or 2 points at best) to the individual components. The usefulness of pursuing a better statistical prediction in spite of reduced practicality—the main advantage of these easy-to-use scores—remains, however, debatable. A novelty here, however, is that the combination of non-duplicated components of the two scores had the highest predictive power for mortality, confirming on the one hand that both ischemic and bleeding events can predict—and in part trigger—the frequent occurrence of death in anticoagulated AF; but also that such components predict triggers of death not easily attributed to thromboembolism or bleeding. The ability to predict such occurrences may allow the focusing on treatment of modifiable risk factors, in addition to only focusing on thrombosis and bleeding, in such populations. Because of the widespread use of the CHA_2_DS_2_-VASc and of the HAS-BLED risk score, a combination of their individual components—as shown here—might be useful to risk-stratify patients in epidemiological surveys and prospective trials, as well as, possibly, to better allocate incremental preventive measures.

## 5. Limitations

Although recruitment of consecutive patients at each center was mandatory in PREFER in AF, we cannot exclude biases in patient enrollment and selection, as well as in treatment decisions. Patients excluded from the study sample were fairly numerous; therefore, a limitation is the relatively frequent lack of reporting of risk scores/missing values in the overall registry. Overall, mean age of the study sample was slightly older than in the non-study sample, but the different distribution of other factors (more use of VKA and higher prevalence of CKD) suggests that patient cases included were perhaps more severe than patients excluded. Such considerations limit, in any case, the generalizability of the findings to the unselected PREFER in AF population. Moreover, mortality data could not exactly reflect real mortality, because data were extracted from the comments section of the electronic case-report forms, making death reporting potentially less accurate. However, we estimated that errors in this largely undisputed outcome are unlikely to be numerically relevant. The investigators’ diligence on reporting adverse events was here indeed crucial for the accuracy of data collection. As for model analyses, there were a couple of limitations when evaluating predictive ability using discrimination and calibration measures. Our study was aimed at mainly investigating the predictive power of the CHA_2_DS_2_-VASc and HAS-BLED risk scores, as well as their combination, on mortality, especially compared to the separate risk scores, therefore no re-calibration of coefficients was conducted. We also deliberately did not venture in analyzing causes of death, likely unprecise and not sufficiently controlled in the registry. The relative shortness of the study follow-up is a further limit with a focus on mortality, but data presented here are the first focusing on this endpoint in anticoagulated patients in attempts at improving prediction with a combination of the two most widely used scores. Finally, at the time of the PREFER in AF registry, most patients with AF were treated with VKAs, which does not reflect the current prevalent treatment with NOACs. Whether such limitations affect current applicability of our findings is likely minor, considering the lack of a biologic plausibility (we do not suspect differences in risk prediction once adequate anticoagulation is ensured), and the reassuring results of our sensitivity analyses. The usefulness of the newly proposed combination score should be now, however, further tested in independent cohorts.

## 6. Conclusions

Mortality is an important component, so far insufficiently underscored, of the risk connected with AF. The CHA_2_DS_2_-VASc and the HAS-BLED scores both predict mortality in AF, and a combination of all their components increases prediction significantly. Such combination may be clinically useful. Until now, the main focus of research in AF has been stroke/SSE and bleeding. The availability of robust, real-world data to inform on patients’ risk of death, now numerically more relevant than stroke/SEE in well-anticoagulated patients, will help to better identify strategies to further improve on AF outcomes.

## Figures and Tables

**Table 1:** Patients’ characteristics at baseline.

		***n***	**%**
**Patients**		5209	100.0
**Age, mean (SD)**		71.76	
**Male gender**		3145	60.4
**Country**			
	France	1264	24.3
	DACH	543	10.4
	Italy	1088	20.9
	Spain	1399	26.9
	UK	915	17.6
**Education**			
	Primary school	2500	48.0
	Secondary school	1651	31.7
	University or above	726	13.9
	Missing	332	6.4
**AF Type**			
	Paroxysmal	1499	28.8
	Persistent	1239	23.8
	Long-standing persistent	391	7.5
	Permanent	2070	39.7
	MIssing	10	0.2
**Medication at Baseline Visit**			
	NOAC	305	5.9
	VKA	3548	68.1
	Antiplatelet	540	10.4
	VKA+Antiplatelet	539	10.4
	Neither NOAC nor VKA nor Antiplatelet	277	5.3
**Medication at Follow-up visits**			
	NOAC	637	12.2
	VKA	3284	63.0
	Antiplatelet	389	7.5
	VKA + Antiplatelet	306	5.9
	Neither NOAC nor VKA nor Antiplatelet	593	11.4
**CHA_2_DS_2_VASc**			
	0	233	4.5
	1	517	9.9
	2	823	15.8
	3	1138	21.9
	4	1176	22.6
	5	722	13.9
	6	376	7.2
	7	151	2.9
	8	63	1.2
	9	10	0.2
**HAS-BLED**			
	0	425	8.2
	1	1264	24.3
	2	1841	35.3
	3	1173	22.6
	4	396	7.6
	5	94	1.8
	6	14	0.3
	7	2	0.0
**Additional comorbities**			
		5209	100.0
	Congestive heart failure	1546	29.7
	Hypertension	3726	71.5
	Diabetes mellitus	1181	22.7
	Stroke/TIA	832	16.0
	Vascular disease	1177	22.6
	Renal function	696	13.4
	Liver function	103	2.0
	Stroke	475	9.1
	Bleeding	244	4.7
	Labile INR	692	13.3
	Drug	1387	26.6
	Alcohol	130	2.5

Abbreviations: SD = standard deviation; DACH = Germany, Austria, and Switzerland; AF = atrial fibrillation; NOAC = non-vitamin K antagonist oral anticoagulant; VKA = vitamin K antagonist; CHA2DS2-VASc = congestive heart failure, hypertension, age 75 years [doubled], diabetes, stroke/transient ischemic attack/thromboembolism [doubled], vascular disease [prior myocardial infarction, peripheral artery disease, or aortic plaque], age 65–75 years, sex category [female]; HAS-BLED = hypertension, abnormal renal/liver function, stroke, bleeding history or predisposition, labile international normalized ratio [INR], elderly [age ≥ 65 years], drugs/alcohol concomitantly; TIA = transient ischemic attack.

**Table 2:** Logit regression results with CHA2DS2-VASc and HAS-BLED combined.

		Coefficient	*p*-Value	Bias	95% CI (Bias-Corrected)		Log-Likelihood	Homes-LEWESHOW	C-Statistic	95% CI (Bias-Corrected)	
							−522.351	1.000	0.731	0.681	0.778
**Stroke/Systemic Embolic Events**											
	Congestive heart failure	0.643	0.002	0.015	0.234	1.038					
	Hypertension	−0.082	0.714	−0.016	−0.467	0.362					
	Age >75 years	0.534	0.089	0.023	−0.059	1.190					
	Diabetes mellitus	0.053	0.811	−0.026	−0.403	0.446					
	Stroke/transient ischemic attack	1.017	0.000	0.011	0.625	1.308					
	Vascular disease	−0.344	0.144	0.017	−0.840	0.071					
	Age 65 to 74 years	0.037	0.917	0.022	−0.634	0.576					
	Sex category	0.283	0.218	0.003	−0.231	0.648					
	Abnormal renal function	−0.193	0.522	−0.016	−0.926	0.327					
	Abnormal liver function	1.014	0.010	−0.041	0.087	1.686					
	Bleeding	0.375	0.345	−0.117	−0.282	1.019					
	Labile INR	0.920	0.000	−0.004	0.460	1.237					
	Drug	0.872	0.000	−0.024	0.494	1.312					
	Alcohol	0.690	0.183	−0.020	−0.502	1.551					
	Constant	−5.079	0.000	−0.059	−5.740	−4.328					
**Major bleeding**							−628.686	0.999	0.702	0.659	0.747
	Congestive heart failure	−0.138	0.487	−0.005	−0.581	0.221					
	Hypertension	0.030	0.887	0.011	−0.353	0.472					
	Age >75 years	0.661	0.015	0.035	0.066	1.119					
	Diabetes mellitus	−0.134	0.498	−0.037	−0.579	0.216					
	Stroke/transient ischemic attack	−0.136	0.582	0.022	−0.546	0.388					
	Vascular disease	0.637	0.001	−0.015	0.299	1.069					
	Age 65 to 74 years	−0.107	0.708	0.020	−0.618	0.368					
	Sex category	0.035	0.868	−0.016	−0.305	0.453					
	Abnormal renal function	0.541	0.006	0.032	0.264	1.002					
	Abnormal liver function	0.890	0.022	−0.045	−0.005	1.654					
	Bleeding	1.425	0.000	0.018	0.947	1.804					
	Labile INR	0.417	0.059	0.007	0.119	0.937					
	Drug	0.219	0.263	−0.013	−0.043	0.677					
	Alcohol	0.904	0.023	−0.077	0.174	1.527					
	Constant	−4.428	0.000	−0.061	−4.976	−3.905					
**Mortality**							−649.047	0.233	0.740	0.699	0.780
	Congestive heart failure	0.802	0.000	0.001	0.365	1.075					
	Hypertension	−0.453	0.013	0.015	−0.811	−0.129					
	Age >75 years	1.051	0.000	0.032	0.460	1.476					
	Diabetes mellitus	0.246	0.222	−0.022	−0.119	0.684					
	Stroke/transient ischemic attack	0.070	0.743	0.018	−0.432	0.399					
	Vascular disease	0.082	0.705	−0.028	−0.335	0.459					
	Age 65 to 74 years	0.229	0.522	0.041	−0.627	0.825					
	Sex category	0.010	0.958	−0.032	−0.365	0.333					
	Abnormal renal function	0.969	0.000	0.032	0.481	1.269					
	Abnormal liver function	1.003	0.001	−0.032	0.399	1.602					
	Bleeding	0.420	0.152	−0.047	−0.229	0.829					
	Labile INR	0.111	0.661	−0.009	−0.411	0.539					
	Drug	0.259	0.178	0.003	−0.080	0.593					
	Alcohol	0.126	0.812	−0.120	−0.918	0.945					
	Constant	−4.625	0.000	−0.061	−5.200	−4.130					

Abbreviations: CHA_2_DS_2_-VASc = congestive heart failure, hypertension, age ≥75 years [doubled], diabetes, stroke/transient ischemic attack/thromboembolism [doubled], vascular disease [prior myocardial infarction, peripheral artery disease, or aortic plaque], age 65–74 years, sex category [female]; HAS-BLED = hypertension, abnormal renal/liver function, stroke, bleeding history or predisposition, labile international normalized ratio [INR], elderly [age ≥65 years], drugs/alcohol concomitantly.

**Table 3:** Evaluation of the predictive ability of the prediction models for the detection of stroke/SSE, major bleeding and mortality using C-index and integrated discrimination improvement indices.

Outcome		C-Statistic	95% CI (Bias-Corrected)		*p*-Value	IDI, %	*p*-Value
**Stroke/SSE**							
	CHA_2_DS_2_-VASc	0.687	0.638	0.730	REF		
	CHA_2_DS_2_-VASc + HAS-BLED	0.731	0.681	0.778	0.010	3.11	0.000
	HAS-BLED	0.670	0.613	0.719	REF		
	CHA_2_DS_2_-VASc + HAS-BLED	0.731	0.681	0.778	0.001	1.46	0.000
**Major bleeding**							
	CHA_2_DS_2_-VASc	0.626	0.579	0.672	REF	2.11	0.000
	CHA_2_DS_2_-VASc + HAS-BLED	0.702	0.659	0.747	0.000		
	HAS-BLED	0.650	0.600	0.698	REF	0.88	0.000
	CHA_2_DS_2_-VASc + HAS-BLED	0.702	0.659	0.747	0.002		
**Mortality**							
	CHA_2_DS_2_-VASc	0.707	0.666	0.744	REF		
	CHA_2_DS_2_-VASc + HAS-BLED	0.740	0.700	0.779	0.005	1.79	0.000
	HAS-BLED	0.646	0.598	0.691	REF		
	CHA_2_DS_2_-VASc + HAS-BLED	0.740	0.700	0.779	0.000	1.26	0.000

Abbreviations: SSE: Systemic Embolic Events; REF; reference model; IDI, integrated discriminating improvement index; CHA2DS2-VASc=congestive heart failure, hypertension, age ≥75 years [doubled], diabetes, stroke/transient ischemic attack/thromboembolism [doubled], vascular disease [prior myocardial infarction, peripheral artery disease, or aortic plaque], age 65–74 years, sex category [female]; HAS-BLED=hypertension, abnormal renal/liver function, stroke, bleeding history or predisposition, labile international normalized ratio [INR], elderly [age ≥65 years], drugs/alcohol concomitantly.

## Supplementary Materials

The following are available online at https://www.mdpi.com/2077-0383/9/12/3987/s1. Table S1. Logit regression result with CHA_2_DS_2_-VASc scores as explanatory variables; Table S2. Logit regression result with HAS-BLED scores as explanatory variables; Table S3. Comparison C-statistic in subjects treated with NOACs vs. total population CHA_2_DS_2_-VASc scores as explanatory variables; Table S4. Comparison C-statistic of the CHA_2_DS_2_-VASc score, the HAS-BLED score and their combination for Stroke/Systemic Embolic Events (SEE), Major Bleeding and Mortality, broken down according to the type of atrial fibrillation—paroxysmal, persistent, long-term persistent and permanent; Table S5: Previous studies of CHA_2_DS_2_-VASc and HAS-BLED scores to predict mortality; online Supplemental References.

## Abbreviations

AF	Atrial fibrillation
ATRIA	AnTicoagulation and Risk factors In Atrial fibrillation
AUC	Area under the curve
CHADS_2_	Congestive heart failure, hypertension, age ≥ 75 years, diabetes, stroke/transient ischemic attack/thromboembolism [doubled]
CHA_2_DS_2_-VASc	Congestive heart failure, hypertension, age ≥ 75 years [doubled], diabetes, stroke/transient ischemic attack/thromboembolism [doubled], vascular disease [prior myocardial infarction, peripheral artery disease, or aortic plaque], age 65–75 years, sex category [female]
CRUSADE	Can Rapid risk stratification of Unstable angina patients Suppress ADverse outcomes with Early implementation of the ACC/AHA guidelines
DACH	Germany (Deutschland), Austria, Switzerland (Confederatio Helvetica)
eCRF	Electronic case report form
GOF	Hosmer and Lemeshow goodness-of-fit test
GRACE	Global Registry of Acute Coronary Events
HAS-BLED	hypertension, abnormal renal/liver function, stroke, bleeding history or predisposition, labile international normalized ratio [INR], elderly [age ≥ 65 years], drugs/alcohol concomitantly
IDI	Integrated discrimination improvement
ISTH	International Society on Thrombosis and Haemostasis
NRI	Net reclassification improvement
NOAC	non-vitamin K antagonist oral anticoagulant
PREFER in AF	Prevention of Thromboembolic Events—European Registry in Atrial Fibrillation” study
SD	Standard deviation of the mean
SEE	Systemic embolic event
TIA	Transient ischemic attack
TIMI	Thrombolysis In Myocardial Infarction
VKA	Vitamin K antagonist
