# Supplementary material for: Mortality Prediction of the CHA2DS2-VASc Score, the HAS-BLED Score, and Their Combination in Anticoagulated Patients with Atrial Fibrillation
Source: J Clin Med. 2020 Dec 9;9(12):3987. doi: 10.3390/jcm9123987 (PMC7764787; doi:10.3390/jcm9123987)
Supplement: Supplementary file 1 [file jcm-09-03987-s001.pdf]

Predictive power for mortality of the CHA_2DS_2-VASc score, the HAS-BLED score and their combination in anticoagulated patients with atrial fibrillation

Running title: Risk scores and mortality in atrial fibrillation

Doralisa Morrone, MD, PhD[1*], Sonja Kroep, PhD[2*], Fabrizio Ricci, MD[3], Giulia Renda, MD, PhD[3], Giuseppe Patti, MD[4], Paulus Kirchhof, MD[5,6], Ling-Hsiang Chuang, PhD[2], Ben van Hout, MD[2,7] and Raffaele De Caterina, MD, PhD[1,8]

*Doralisa Morrone and Sonja Kroep share first authorship

[1]Institute of Cardiology, University of Pisa; [2]Pharmerit International, Rotterdam, the Netherlands; [3]Institute of Cardiology and Center of Excellence on Aging, G. d'Annunzio University – Chieti-Pescara, Italy; [4]University of Eastern Piedmont – Novara, Italy; [5]University of Birmingham Institute of Cardiovascular Sciences, University of Birmingham, UHB and SWBH NHS Trusts, Birmingham, UK, [6]University Heart and Vascular Center Hamburg, Hamburg, Germany; [7]University of Sheffield, Sheffield, UK; [8]Fondazione VillaSerena per la Ricerca, Città Sant'Angelo, Pescara, Italy

SUPPLEMENTARY MATERIALS

(Abbreviations, Highlights, Supplemental Tables, Supplemental references)

Correspondence:

Raffaele De Caterina, MD PhD,

Cardiovascular and Thoracic Department, University of Pisa

C/o University Cardiology Division - Pisa University Hospital

Via Paradisa, 2 - 56124 Pisa, Italy

Tel: +39-050-221-1848 - E-mail: raffaele.decaterina@unipi.it

ABBREVIATIONS (in alphabetical order)

AF = Atrial fibrillation

ATRIA = AnTicoagulation and Risk factors In Atrial fibrillation

AUC = area under the curve

$CHADS_2$ = congestive heart failure, hypertension, age≥75 years, diabetes, stroke/transient ischemic attack/thromboembolism [doubled

CHA_2DS_2-VASc = congestive heart failure, hypertension, age≥75 years [doubled], diabetes, stroke/transient ischemic attack/thromboembolism [doubled], vascular disease [prior myocardial infarction, peripheral artery disease, or aortic plaque], age 65-75 years, sex category [female]

CRUSADE = Can Rapid risk stratification of Unstable angina patients Suppress ADverse outcomes with Early implementation of the ACC/AHA guidelines

DACH = Germany (Deutschland), Austria, Switzerland (Confederatio Helvetica)

eCRF = electronic case report form

GOF = Hosmer and Lemeshow goodness of fit test

GRACE = Global Registry of Acute Coronary Events

HAS-BLED = hypertension, abnormal renal/liver function, stroke, bleeding history or predisposition, labile international normalized ratio [INR], elderly [age ≥65 years], drugs/alcohol concomitantly

IDI = integrated discrimination improvement

ISTH = International Society on Thrombosis and Haemostasis

NRI = net reclassification improvement

NOAC = non-vitamin K antagonist oral anticoagulant

PREFER in AF = Prevention of Thromboembolic Events – European Registry in Atrial Fibrillation" study

SD = standard deviation of the mean

SEE = systemic embolic event

TIA = transient ischemic attack

TIMI = Thrombolysis In Myocardial Infarction

VKA = vitamin K antagonist

HIGHLIGHTS

- Atrial fibrillation (AF) is associated with increased mortality, but predictors of mortality are poorly characterized.
- We here investigated the predictive power of the CHA_2DS_2-VASc and the HAS-BLED score, commonly used to assess the risk of stroke and bleeding, for mortality in patients with AF in a large contemporary registry.
- In the PREFER in AF prospective, real-world registry including AF patients across 7 European countries in 5,209 AF patients, the average 1-year mortality was 3.1%.
- We found strong gradients between all examined outcomes (mortality, stroke and systemic embolic events and major bleeding) for both the CHA_2DS_2-VASc and the HAS-BLED risk scores, with similar c-statistics.
- The predictive power with both scores combined, removing overlapping components, was higher than individual scores separately. Such combination may thus be clinically useful.

Table S1. Logit regression result with CHA_2DS_2-VASc scores as explanatory variables

		Coefficient	P-value	Bias	95% CI (Bias-	corrected)	Log-likelihood	Homes-Leweshow	C-statistic	95% CI (Bias-	corrected)
**Stroke/SSE**							-560.76	0.014	0.584	0.536	0.637
	Total score	0.307	0.00	0.00	0.23	0.46					
	Constant	-4.918	0.00	-0.01	-5.51	-4.52					
**Stroke/SSE**							-551.266	0.691	0.687*	0.638	0.730
	Congestive heart failure	0.775	0.00	-0.02	0.51	1.18					
	Hypertension	-0.100	0.66	0.02	-0.50	0.37					
	Age >75 years	0.458	0.14	0.00	-0.06	1.14					
	Diabetes mellitus	0.079	0.73	-0.02	-0.33	0.53					
	Stroke/TIA	1.026	0.00	0.00	0.52	1.38					
	Vascular disease	-0.120	0.61	0.04	-0.69	0.27					
	Age 65 to 74 years	0.043	0.89	-0.01	-0.53	0.88					
	Sex category	0.276	0.16	0.03	-0.16	0.65					
	Constant	-4.571	0.00	-0.06	-5.27	-4.09					
**Major bleeding**							-668.392	0.269	0.520	0.478	0.567
	Total score	0.188	0.000	-0.002	0.119	0.271					
	Constant	-4.219	0.000	-0.015	-4.651	-3.882					
**Major bleeding**							-656.538	0.446	0.626*	0.579	0.672
	Congestive heart failure	0.050	0.776	0.003	-0.353	0.342					
	Hypertension	0.072	0.718	-0.020	-0.212	0.621					
	Age >75 years	0.685	0.012	0.019	0.199	1.114					
	Diabetes mellitus	-0.013	0.946	0.007	-0.351	0.301					
	Stroke/TIA	-0.028	0.902	-0.041	-0.457	0.488					
	Vascular disease	0.726	0.000	0.020	0.316	1.088					
	Age 65 to 74 years	-0.081	0.791	0.010	-0.611	0.454					
	Sex category	-0.024	0.901	0.013	-0.397	0.341					
	Constant	-4.148	0.000	-0.041	-4.567	-3.594					
**Mortality**							-695.570	0.039	0.559	0.513	0.604
	Total score	0.280	0.000	0.003	0.208	0.372					
	Constant	-4.523	0.000	-0.036	-4.867	-4.227					
**Mortality**							-668.911	0.005	0.707*	0.666	0.744
	Congestive heart failure	0.962	0.000	0.020	0.581	1.226					
	Hypertension	-0.416	0.020	0.028	-0.826	-0.130					
	Age >75 years	1.180	0.000	0.085	0.722	1.693					
	Diabetes mellitus	0.368	0.034	-0.030	0.103	0.872					
	Stroke/TIA	0.090	0.670	0.008	-0.294	0.491					
	Vascular disease	0.209	0.236	-0.019	-0.115	0.562					
	Age 65 to 74 years	0.270	0.426	0.084	-0.364	0.951					
	Sex category	-0.018	0.929	0.013	-0.454	0.332					
	Constant	-4.481	0.000	-0.134	-4.999	-4.044					

Abbreviations: SSE: Systemic Embolic Events; TIA: Transient Ischemic Attack; CHA_2DS_2-VASc=congestive heart failure, hypertension, age≥75 years [doubled], diabetes,
* Significant improvement

Table S2. Logit regression result with HAS-BLED scores as explanatory variables

		Coefficient	P-value	Bias	95% CI (Bias-corrected)		Log-likelihood	Homes-Leweshow	C-statistic	95% CI (Bias-corrected)	
**Stroke/SEE**							-548.924	0.432	0.571	0.512	0.631
	Total score	0.582	0.000	-0.005	0.453	0.738					
	Constant	-5.140	0.000	0.013	-5.534	-4.806					
**Stroke/SEE**							-536.495	0.576	0.670*	0.613	0.719
	Hypertension	0.042	0.823	-0.016	-0.314	0.432					
	Renal function	-0.007	0.977	-0.077	-0.448	0.436					
	Liver function	1.119	0.003	0.055	0.316	1.750					
	Stroke	0.962	0.000	-0.013	0.504	1.370					
	Bleeding	0.356	0.379	-0.040	-0.561	0.888					
	Labile INR	1.006	0.000	-0.018	0.704	1.490					
	Age> 65 years	0.514	0.049	-0.003	0.085	1.042					
	Drug	0.809	0.000	0.011	0.505	1.169					
	Alcohol	0.626	0.174	-0.061	-0.201	1.566					
	Constant	-4.902	0.000	-0.002	-5.474	-4.437					
**Major bleeding**							-653.214	0.594	0.534	0.486	0.584
	Total score	0.472	0.000	-0.006	0.301	0.586					
	Constant	-4.631	0.000	0.000	-5.024	-4.202					
**Major bleeding**							-640.898	0.156	0.650*	0.600	0.698
	Hypertension	0.081	0.620	-0.007	-0.230	0.405					
	Renal function	0.662	0.002	-0.025	0.266	1.051					
	Liver function	0.864	0.039	-0.089	0.019	1.681					
	Stroke	0.054	0.858	-0.004	-0.567	0.490					
	Bleeding	1.374	0.000	-0.001	1.005	1.791					
	Labile INR	0.401	0.052	-0.033	0.027	0.786					
	Age> 65 years	0.531	0.023	0.015	0.068	1.024					
	Drug	0.335	0.098	0.016	-0.097	0.718					
	Alcohol	0.791	0.029	-0.031	-0.164	1.289					
	Constant	-4.491	0.000	-0.019	-4.948	-3.942					
**Mortality**							-697.432	0.250	0.504	0.454	0.554
	Total score	0.397	0.000	-0.007	0.322	0.520					
	Constant	-4.361	0.000	-0.007	-4.784	-4.114					
**Mortality**							-671.598	0.772	0.646*	0.598	0.691
	Hypertension	-0.337	0.039	-0.008	-0.681	-0.070					
	Renal function	1.246	0.000	-0.020	1.019	1.568					
	Liver function	1.154	0.002	-0.048	0.475	1.850					
	Stroke	0.029	0.926	-0.033	-0.493	0.791					
	Bleeding	0.423	0.166	-0.031	-0.170	0.995					
	Labile INR	0.262	0.196	-0.025	-0.168	0.569					
	Age> 65 years	0.825	0.003	0.119	0.293	1.297					
	Drug	0.317	0.027	0.001	0.066	0.586					
	Alcohol	0.128	0.814	-0.142	-1.345	0.998					
	Constant	-4.447	0.000	-0.090	-4.899	-4.066					

Abbreviations: HAS-BLED=hypertension, abnormal renal/liver function, stroke, bleeding history or predisposition, labile international normalized ratio [INR], elderly [age ≥65 years], drugs/alcohol concomitantly.

* Significant improvement

Table S3. Comparison C-statistic in subjects treated with NOACs vs total population

CHA_2DS_2-VASc scores as explanatory variables

			C-statistic	95% CI (Bias-corrected)	
**Stroke/SEE**	Total score	NOAC subgroup	0.466	0.349	0.590
		Total population	0.584	0.536	0.637
	Individual components	NOAC subgroup	0.662	0.525	0.778
		Total population	0.687	0.638	0.730
**Major bleeding**	Total score	NOAC subgroup	0.491	0.393	0.585
		Total population	0.520	0.478	0.567
	Individual components	NOAC subgroup	0.674	0.586	0.760
		Total population	0.626	0.579	0.672
**Mortality**	Total score	NOAC subgroup	0.596	0.419	0.752
		Total population	0.559	0.513	0.604
	Individual components	NOAC subgroup	0.829	0.695	0.940
		Total population	0.707	0.666	0.744

HAS-BLED scores as explanatory variables

			C-statistic	95% CI (Bias-corrected)	
**Stroke/SEE**	Total score	NOAC subgroup	0.411	0.280	0.553
		Total population	0.571	0.512	0.631
	Individual components	NOAC subgroup	0.609	0.445	0.747
		Total population	0.670	0.613	0.719
**Major bleeding**	Total score	NOAC subgroup	0.536	0.420	0.663
		Total population	0.534	0.486	0.584
	Individual components	NOAC subgroup	0.617	0.511	0.730
		Total population	0.650	0.600	0.698
**Mortality**	Total score	NOAC subgroup	0.544	0.318	0.781
		Total population	0.504	0.454	0.554
	Individual components	NOAC subgroup	0.635	0.369	0.852
		Total population	0.646	0.598	0.691

CHA_2DS_2-VASc and HAS-BLED combined as explanatory variables

			C-statistic	95% CI (Bias-corrected)	
**Stroke/SEE**	Individual components	NOAC subgroup	0.689	0.569	0.803
		Total population	0.731	0.681	0.778
**Major bleeding**	Individual components	NOAC subgroup	0.725	0.630	0.822
		Total population	0.702	0.659	0.747
**Mortality**	Individual components	NOAC subgroup	0.863	0.737	0.967
		Total population	0.740	0.699	0.780

Abbreviations: NOAC: non-vitamin K antagonist oral anticoagulant; SSE: Systemic Embolic Events; CHA_2DS_2-VASc=congestive heart failure, hypertension, age >75 years [doubled], diabetes, stroke/transient ischemic attack/thromboembolism [doubled], vascular disease [prior myocardial infarction, peripheral artery disease, or aortic plaque], age 65-75 years, sex category [female]; HAS-BLED=hypertension, abnormal renal/liver function, stroke, bleeding history or predisposition, labile international normalized ratio [INR], elderly [age ≥65 years], drugs/alcohol concomitantly.

Table S4. Comparison C-statistic of the CHA_2DS_2-VASc score, the HAS-BLED score and their combination for Stroke/Systemic Embolic Events (SEE), Major Bleeding and Mortality, broken down according to the type of atrial fibrillation - paroxysmal, persistent, long-term persistent and permanent.

Paroxysmal atrial fibrillation

Outcome		C-statistic	95% CI (Bias-corrected)	
**Stroke/SSE**				
	CHA_2DS_2-VASc	0.619	0.520	0.718
	CHA_2DS_2-VASc + HAS-BLED	0.614	0.510	0.718
	HAS-BLED	0.543	0.438	0.648
	CHA_2DS_2-VASc + HAS-BLED	0.614	0.510	0.718
**Major bleeding**				
	CHA_2DS_2-VASc	0.547	0.461	0.633
	CHA_2DS_2-VASc + HAS-BLED	0.600	0.502	0.698
	HAS-BLED	0.577	0.483	0.671
	CHA_2DS_2-VASc + HAS-BLED	0.600	0.502	0.698
**Mortality**				
	CHA_2DS_2-VASc	0.739	0.632	0.845
	CHA_2DS_2-VASc + HAS-BLED	0.732	0.620	0.843
	HAS-BLED	0.631*	0.498	0.763
	CHA_2DS_2-VASc + HAS-BLED	0.732	0.620	0.843

Persistent atrial fibrillation

Outcome		C-statistic	95% CI (Bias-corrected)	
**Stroke/SSE**				
	CHA_2DS_2-VASc	0.775	0.693	0.858
	CHA_2DS_2-VASc + HAS-BLED	0.825	0.747	0.904
	HAS-BLED	0.776	0.681	0.871
	CHA_2DS_2-VASc + HAS-BLED	0.825	0.747	0.904
**Major bleeding**				
	CHA_2DS_2-VASc	0.739	0.651	0.827
	CHA_2DS_2-VASc + HAS-BLED	0.750	0.661	0.838
	HAS-BLED	0.692*	0.578	0.805
	CHA_2DS_2-VASc + HAS-BLED	0.750	0.661	0.838
**Mortality**				
	CHA_2DS_2-VASc	0.727	0.639	0.816
	CHA_2DS_2-VASc + HAS-BLED	0.776	0.688	0.865
	HAS-BLED	0.739	0.642	0.837
	CHA_2DS_2-VASc + HAS-BLED	0.776	0.688	0.865

Long-standing persistent atrial fibrillation

Outcome		C-statistic	95% CI (Bias-corrected)	
**Stroke/SSE**				
	CHA_2DS_2-VASc	0.654	0.344	0.963
	CHA_2DS_2-VASc + HAS-BLED	0.701	0.447	0.954
	HAS-BLED	0.609	0.316	0.903
	CHA_2DS_2-VASc + HAS-BLED	0.701	0.447	0.954
**Major bleeding**				
	CHA_2DS_2-VASc	0.554*	0.360	0.748
	CHA_2DS_2-VASc + HAS-BLED	0.765	0.533	0.997
	HAS-BLED	0.848	0.706	0.991
	CHA_2DS_2-VASc + HAS-BLED	0.765	0.533	0.997
**Mortality**				
	CHA_2DS_2-VASc	0.620	0.472	0.768
	CHA_2DS_2-VASc + HAS-BLED	0.740	0.638	0.841
	HAS-BLED	0.667	0.519	0.815
	CHA_2DS_2-VASc + HAS-BLED	0.740	0.638	0.841

Permanent atrial fibrillation

Outcome		C-statistic	95% CI (Bias-corrected)	
**Stroke/SSE**				
	CHA_2DS_2-VASc	0.686*	0.617	0.756
	CHA_2DS_2-VASc + HAS-BLED	0.760	0.688	0.832
	HAS-BLED	0.691*	0.604	0.778
	CHA_2DS_2-VASc + HAS-BLED	0.760	0.688	0.832
**Major bleeding**				
	CHA_2DS_2-VASc	0.633*	0.565	0.701
	CHA_2DS_2-VASc + HAS-BLED	0.731	0.668	0.794
	HAS-BLED	0.655*	0.580	0.729
	CHA_2DS_2-VASc + HAS-BLED	0.731	0.668	0.794
**Mortality**				
	CHA_2DS_2-VASc	0.665	0.609	0.720
	CHA_2DS_2-VASc + HAS-BLED	0.698	0.641	0.754
	HAS-BLED	0.584*	0.512	0.656
	CHA_2DS_2-VASc + HAS-BLED	0.698	0.641	0.754

* denotes statistically different values compared with the following value in the column.

Table 5S: Previous studies of CHA$_2$DS$_2$-VASc and HAS-BLED scores to predict mortality

First Author	Year	Reference	Score(s) studied	No. of Patients	Type of patients studied	Main findings
Gallego, P.	2012	1	HAS-BLED	965	Atrial fibrillation	The HAS-BLED score shows some predictive value for cardiovascular events and mortality in anticoagulated patients with AF, consistent with the relationship between thrombosis and bleeding.
Padjen, V.	2013	2	CHA2DS2 -VASc, HAS-BLED	787	Atrial fibrillation	HAS-BLED was found to have an independent predictive value on the occurrence of sICH regardless of the treatment (thrombolysis or conservative therapy). The CHA2DS2-VASc score was inversely related to the favourable outcome in the univariate analysis (OR 0.80; 95% CI 0.65-0.99, P = 0.042)
Capodanno, D.	2015	3	CHA2DS2 -VASc, HAS-BLED	1437	Atrial fibrillation	In patients without AF undergoing PCI and discharged on dual antiplatelet therapy, the HAS-BLED score performed better than the CHA2DS2-VASc for the prediction of MACE. Although both scores predict MACE, their discrimination was modest. Conversely, both scores did not significantly predict major bleeding in non-AF patients undergoing PCI.
Sadeghi, R.	2015	4	CHA2DS2-VASc	900	Ischemic stroke	Patients with atrial fibrillation hospitalized with ischemic stroke showed an important absolute risk of further stroke and early mortality. Despite substantiated advantages of warfarin prophylaxis, the application of the score is still very limited.
García-Fernández A	2016	5	HAS-BLED	406	Atrial fibrillation	In AF patients undergoing electrical cardioversion, major bleeding episodes and mortality were independently associated with poor quality control of anticoagulation and previous bleeding. The HAS-BLED score successfully predicted major bleeding and mortality.
Yoshihisa, A.	2016	6	CHA2DS2-VASc	1011	Atrial fibrillation and heart failure	A CHA2DS2-VASc score 7-9 was an independent predictor of all-cause mortality in heart failure (HF): for all HF patients: hazard ratio (HR) 1.822, P = 0.011; or HF patients with AF: HR 1.951, P = 0.031; for HF patients without AF: HR 2.215, P = 0.033).
Fox, KAA	2017	7	GARFIELD VS CHA2DS2-VASc	39898	Atrial fibrillation	Performance of the GARFIELD-AF risk tool was superior to CHA2DS2-VASc in predicting stroke and mortality and superior to HAS-BLED for bleeding, overall and in lower risk patients.
Mazzone, C.	2017	8	CHA2DS2-VASc	12599	Patients in sinus rhythm	In a community population of Caucasian arterial hypertension patients in sinus rhythm, CHA2DS2-VASc well stratified for adverse clinical events at a mid-term follow-up with accuracy similar as in atrial fibrillation patients.
Chen, Yung-Lung	2017	9	CHA2DS2-VASc	1311	Heart failure with reduced ejection fraction	CHADS2, CHA2DS2-VASc, and R2CHADS2 scores can be used to predict 1-year all-cause mortality in patients with heart failure with preserved ejection fraction with or without atrial fibrillation. For predicting all-cause mortality in uch patients, the R2CHADS2 is more accurate than CHADS2 and CHA2DS2-VASc.
Graves G	2018	10	CHA2DS2-VASc	10077	Atrial fibrillation	Using the Intermountain Mortality Risk Score (IMRS) jointly with CHA2DS2-VASc in patients with atrial fibrillation improved the prediction of stroke and mortality. For example, in patients at the OAC treatment threshold (CHA2DS2-VASc = 2), the IMRS provided ≈4-fold separation between low and high risk. The IMRS provides an enhancing marker for risk in patients with AF that reflects the underlying systemic nature of this disease that may be considered in combination with the CHA2DS2-VASc score.
Gazova, A.	2019	11	CHA2DS2 VASc	137	Atrial fibrillation	The CHA2DS2-VASc score correctly predicted the patients at high-risk for 3 to 5 years mortality and confirmed its significant predictive value in the patients with atrial fibrillation.
Cioffi, G.	2019	12	CHA2DS2-VASc	414	Inflammatory arthritis	The CHA2DS2 VASc score accurately predicts cardiovascular and non cardiovascular mortality and hospitalization in patients in sinus rhythm with rheumatoid arthritis, ankylosing spondylitis and psoriatic arthritis in the mid term.
Nof, E.	2020	13	CHA2DS2 VASc	1804	Patients with an implanted cardioverter-defibrillator (MADIT Study)	A high CHA2DS2 VASc score can be used to identify patients with mild heart failure who have low risk of ventricular tachyarrhythmias and high morbidity risk or mortality, and may derive a pronounced clinical benefit from cardiac resynchronization therapy without a defibrillator. The authors suggest a possible role for the CHA2DS2 VASc score in device selection among candidates to biventricular pacing.
Karamchandani, k	2020	14	CHA2DS2-VASc	640	New-onset atrial fibrillation	New-onset atrial fibrillation is common in critically ill patients and is associated with high in-hospital mortality. The CHA2DS2-VASc score is not a reliable prognostic marker of in-hospital mortality in such patients. However, the presence of vascular disease in such patients is associated with increased mortality.
Hsieh, MJ	2017	15	HAS-BLED	617	Non-ST elevation myocardial infarction without atrial fibrillation	The HAS-BLED scoring system is similar to the GRACE and CRUSADE scores but better than the TIMI risk score to predict long-term survival outcomes in patients with non-ST elevation myocardial infarction without atrial fibrillation.
Ipek, G.	2016	16	CHA2DS2-VASc	2375	ST-elevation myocardial infarction	The CHA2DS2-VASc score is associated with a higher risk of no-reflow and in-hospital mortality in patients who undergoing primary percutaneous coronary intervention.
Melgaard, L.	2015	17	CHA2DS2-VASc	42987	Heart failure with and without atrial fibrillation	Among patients with incident heart failure, with or without atrial fibrillation, the CHA2DS2-VASc score was predicted ischemic stroke, thromboembolism, and death. The absolute risk of thromboembolic complications was higher among patients without atrial fibrillation compared with patients with concomitant atrial fibrillation at high CHA2DS2-VASc scores. However, the predictive accuracy was modest, and the clinical utility of the CHA2DS2-VASc score in patients with heart failure remains to be determined.
Mitchell, L.B.	2014	18	CHA2DS2-VASc	20970	Acute coronary syndrome without atrial fibrillation (Alberta Provincial Project for Outcomes Assessment in Coronary Heart disease (APPROACH) prospective registry)	In patients with acute coronary syndrome but no atrial fibrillation, the CHADS2 and CHA2DS2-VASc scores predict ischaemic stroke and transient ischemic attack with similar accuracy as in historical populations with non-valvular AF, but with lower absolute event rates.
Oldgren, J.	2011	19	CHADS2	18112	Atrial fibrillation	Higher CHADS2 scores were associated with increased risks for stroke or systemic embolism, bleeding, and death in patients with atrial fibrillation receiving oral anticoagulants.
Onuk, T.	2017	20	CHA2DS2-VASc	277	Acute pulmonary embolism without atrial fibrillation	A CHA2DS2-VASc score ≥4 predicts a 16.8 times higher risk of eath compared to patients with CHA2DS2-VASc score = 0.
Paoletti Perini, A.	2014	21	CHA2DS2-VASc	559	Patients candidates to cardiac resynchronization therapy	In CRT-D patients with cardiac resynchronization therapy and implanted defibrillator (CRT-D), a pre-implant CHA2DS2-VASc score is an independent predictor of major clinical events at a 30-month follow-up.
Patti, G.	2019	22	CHA2DS2-VASc	610	Atrial fibrillation	In a population-based study on diabetic patients without atrial fibrillation, the CHA2 DS2 -VASc score was an independent predictor of ischemic stroke, coronary events, and overall mortality.
Peacock, W.F.	2017	23	CHA2DS2-VASc	44793	Atrial fibrillation	Patients with higher scores had higher mortality.
Renda, G.	2019	24	CHA2DS2-VASc	22179	Patients with or without new-onset atrial fibrillation	The CHA2DS2VASc score is a sensitive tool for predicting new-onset atrial fibrillation and adverse outcomes in subjects both with and without atrial fibrillation.
Temizer, O.	2017	25	CHA2DS2-VASc	106	Heart failure with reduced ejection fraction	The CHA2DS2-VASc score and functional capacity were the only predictors of 1-year mortality in patients with heart failure with reduced ejection fraction.

Abbreviations: CHADS2=Congestive heart failure , Hypertension,; Age ≥75 years, Diabetes mellitus; Prior Stroke or TIA or Thromboembolism (doubled); CHA2DS2-VASc=congestive heart failure, hypertension, age ≥75 years [doubled], diabetes, stroke/transient ischemic attack/thromboembolism [doubled], vascular disease [prior myocardial infarction,

SUPPLEMENTAL REFERENCES

1. Gallego P, Roldan V, Torregrosa JM, Galvez J, Valdes M, Vicente V, Marin F, Lip GY. Relation of the has-bled bleeding risk score to major bleeding, cardiovascular events, and mortality in anticoagulated patients with atrial fibrillation. Circ Arrhythm Electrophysiol. 2012;5:312-318

2. Padjen V, Jovanovic DR, Leys D, Beslac-Bumbasirevic L. Predicting the outcomes of acute ischaemic stroke in atrial fibrillation: The role of baseline CHADS2, CHA2DS2-VASc and HAS-BLED score values. Acta Cardiol. 2013;68:590-596

3. Capodanno D, Rossini R, Musumeci G, Lettieri C, Senni M, Valsecchi O, Angiolillo DJ, Lip GY. Predictive accuracy of CHA2DS2-VASc and HAS-BLED scores in patients without atrial fibrillation undergoing percutaneous coronary intervention and discharged on dual antiplatelet therapy. Int J Cardiol. 2015;199:319-325

4. Sadeghi R, Parsa Mahjoob M, Asadollahi M, Abbasi Z. Prevalence, main determinants, and early outcome of patients with atrial fibrillation hospitalized with ischemic stroke: Evaluation of the value of risk assessment scores for predicting risk of stroke or major bleeding following anticoagulation therapy. Acta Biomed. 2015;86:162-169

5. García-Fernández A, Marín F, Roldán V, Galcerá-Jornet E, Martínez-Martínez JG, Valdés M, Sogorb F, Lip GY. The HAS-BLED score predicts long-term major bleeding and death in anticoagulated non-valvular atrial fibrillation patients undergoing electrical cardioversion. Int J Cardiol. 2016;217:42-48

6. Yoshihisa A, Watanabe S, Kanno Y, Takiguchi M, Sato A, Yokokawa T, Miura S, Shimizu T, Abe S, Sato T, Suzuki S, Oikawa M, Sakamoto N, Yamaki T, Sugimoto K, Kunii H, Nakazato K, Suzuki H, Saitoh S-I, Takeishi Y. The CHA2DS2-VASc score as a predictor of high mortality in hospitalized heart failure patients. ESC Heart Failure. 2016;3:261-269

7. Fox KAA, Lucas JE, Pieper KS, Bassand JP, Camm AJ, Fitzmaurice DA, Goldhaber SZ, Goto S, Haas S, Hacke W, Kayani G, Oto A, Mantovani LG, Misselwitz F, Piccini JP, Turpie AGG, Verheugt FWA, Kakkar AK. Improved risk stratification of patients with atrial fibrillation: An integrated GARFIELD-AF tool for the prediction of mortality, stroke and bleed in patients with and without anticoagulation. BMJ Open. 2017;7:e017157

8. Mazzone C, Cioffi G, Carriere C, Barbati G, Faganello G, Russo G, Cherubini A, Sinagra G, Zeriali N, Di Lenarda A. Predictive role of CHA2DS2-VASc score for cardiovascular events and death in patients with arterial hypertension and stable sinus rhythm. Eur J Prev Cardiol. 2017;24:1584-1593

9. Chen YL, Cheng CL, Huang JL, Yang NI, Chang HC, Chang KC, Sung SH, Shyu KG, Wang CC, Yin WH, Lin JL, Chen SM. Mortality prediction using CHADS2/ CHA2DS2-VASc scores in systolic heart failure patients with or without atrial fibrillation. Medicine (Baltimore). 2017;96:e8338

10. Graves KG, May HT, Knowlton KU, Muhlestein JB, Jacobs V, Lappé DL, Anderson JL, Horne BD, Bunch TJ. Improving CHA2DS2-VASc stratification of non-fatal stroke and mortality risk using the intermountain mortality risk score among patients with atrial fibrillation. Open Heart. 2018;5:e000907

11. Gažová A, Leddy JJ, Rexová M, Hlivák P, Hatala R, Kyselovič J. Predictive value of CHA2DS2-VASc scores regarding the risk of stroke and all-cause mortality in patients with atrial fibrillation (consort compliant). Medicine (Baltimore). 2019;98:e16560

12. Cioffi G, Viapiana O, Orsolini G, Idolazzi L, Fracassi E, Ognibeni F, Dalbeni A, Gatti D, Carletto A, Fassio A, Rossini M, Giollo A. Usefulness of CHA2DS2-VASc score to predict mortality and hospitalization in patients with inflammatory arthritis. Int J Rheum Dis. 2020;23:106-115

13. Nof E, Kutyifa V, McNitt S, Goldberger J, Huang D, Aktas MK, Spencer R, Goldenberg I, Beinart R. CHA2DS2-VASc score and the risk of ventricular

tachyarrhythmic events and mortality in MADIT-CR. J Am Heart Assoc. 2020;9:e014353

14. Karamchandani K, Schoaps RS, Abendroth T, Carr ZJ, King TS, Bonavia A. CHA2DS2-VASc score and in-hospital mortality in critically ill patients with new-onset atrial fibrillation. J Cardiothorac Vasc Anesth. 2020;34:1165-1171

15. Hsieh MJ, Lee CH, Chen CC, Chang SH, Wang CY, Hsieh IC. Predictive performance of HAS-BLED risk score for long-term survival in patients with non-st elevated myocardial infarction without atrial fibrillation. J Cardiol. 2017;69:136-143

16. Ipek G, Onuk T, Karatas MB, Gungor B, Osken A, Keskin M, Oz A, Tanik O, Hayiroglu MI, Yaka HY, Ozturk R, Bolca O. CHA2DS2-VASc score is a predictor of no-reflow in patients with st-segment elevation myocardial infarction who underwent primary percutaneous intervention. Angiology. 2016;67:840-845

17. Melgaard L, Gorst-Rasmussen A, Lane DA, Rasmussen LH, Larsen TB, Lip GY. Assessment of the CHA2DS2-VASc score in predicting ischemic stroke, thromboembolism, and death in patients with heart failure with and without atrial fibrillation. JAMA. 2015;314:1030-1038

18. Mitchell LB, Southern DA, Galbraith D, Ghali WA, Knudtson M, Wilton SB, investigators A. Prediction of stroke or tia in patients without atrial fibrillation using CHADS2 and CHA2DS2-VASc scores. Heart. 2014;100:1524-1530

19. Oldgren J, Alings M, Darius H, Diener HC, Eikelboom J, Ezekowitz MD, Kamensky G, Reilly PA, Yang S, Yusuf S, Wallentin L, Connolly SJ, Investigators R-L. Risks for stroke, bleeding, and death in patients with atrial fibrillation receiving dabigatran or warfarin in relation to the CHADS2 score: A subgroup analysis of the re-ly trial. Ann Intern Med. 2011;155:660-667, W204

20. Onuk T, Karatas MB, Ipek G, Gungor B, Akyuz S, Canga Y, Uzun AO, Avci, II, Osken A, Kasikcioglu H, Cam N. Higher CHA2DS2-VASc score is associated with increased mortality in acute pulmonary embolism. Clin Appl Thromb Hemost. 2017;23:631-637

21. Paoletti Perini A, Bartolini S, Pieragnoli P, Ricciardi G, Perrotta L, Valleggi A, Vergaro G, Michelotti F, Boggian G, Sassone B, Mascioli G, Emdin M, Padeletti L. CHADS2 and CHA2DS2-VASc scores to predict morbidity and mortality in heart failure patients candidates to cardiac resynchronization therapy. Europace. 2014;16:71-80

22. Patti G, Sticchi A, Pasceri V, Ricci F, Renda G, Hamrefors V, Melander O, Sutton R, Engstrom G, De Caterina R, Fedorowski A. The co-predictive value of a cardiovascular score for CV outcomes in diabetic patients with no atrial fibrillation. Diabetes/Metabolism Research and Reviews. 2019:35(5):e3145 - DOI: 10.1002/dmrr.3145; PMID: 30790434

23. Peacock WF, Tamayo S, Patel M, Sicignano N, Hopf KP, Yuan Z. CHA2DS2-VASc scores and major bleeding in patients with nonvalvular atrial fibrillation who are receiving rivaroxaban. Ann Emerg Med. 2017;69:541-550 e541

24. Renda G, Ricci F, Patti G, Aung N, Petersen SE, Gallina S, Hamrefors V, Melander O, Sutton R, Engstrom G, Caterina R, Fedorowski A. CHA2DS2-VASc and adverse outcomes in middle-aged individuals without atrial fibrillation. Eur J Prev Cardiol. 2019:2047487319868320

25. Temizer O, Acar B, Yayla C, Unal S, Goktug Ertem A, Gucuk Ipek E, Canpolat U, Senturk B, Selcuk H, Selcuk T. The association between CHA2DS2-VASc score and mortality in patients with heart failure with reduced ejection fraction. Acta Cardiol Sin. 2017;33:429-435
